# Comorbidities in hospitalized patients with herpes zoster: an Italian retrospective observational study in the years 2011–2023

**DOI:** 10.1186/s12879-025-12319-x

**Published:** 2025-12-25

**Authors:** Antonella Mattei, Debora Cialfi, Alberto D’Annunzio, Leila Fabiani, Fabiana Fiasca, Giovanni Gabutti

**Affiliations:** 1 Department of Life, Health and Environmental Sciences, University of L’Aquila, 67100 L’Aquila, Italy; 2 SS. Filippo and Nicola Hospital, ASL 1 Avezzano-Sulmona-L’Aquila, 67051 Avezzano, Italy; 3 Working Group “Vaccines and Immunization Policies”, Italian Society of Hygiene, Preventive Medicine and Public Health, 16030 Cogorno, Italy

**Keywords:** Herpes zoster, Comorbidity, Hospitalizations, Epidemiology, Italy

## Abstract

**Background:**

Herpes Zoster (HZ) is caused by the reactivation of the varicella zoster virus (VZV). Although aging is the most well-known risk factor for herpes zoster, little is known about the strength of the association between patient-related characteristics, including comorbidities, and their contribution to an increased risk of mortality, among patients hospitalized for HZ. The aim of this study was to assess hospitalization trends and the strength of this association.

**Methods:**

This retrospective population-based study, conducted among all patients hospitalized with HZ in Italy, between January 1st 2011 and December 31st 2023, analysed Hospital Discharge Records (HDR) reporting the ICD-9-CM codes related to HZ infection. Comorbidities that induce a reduced response of VZV-specific cell-mediated immunity - such as malignant neoplasms, chronic obstructive pulmonary disease, kidney diseases, diabetes mellitus, autoimmune diseases - were considered. Poisson regressions models, adjusted for age classes and sex, were used to identify factors associated with an increased risk of death.

**Results:**

Between 2011 and 2023, 47,933 hospitalizations with HZ were recorded. HZ comorbidities were diagnosed in 27.71% of cases (13,280 hospitalizations). The analysis revealed that the presence of neoplasms (IRR: 3.15; 95% C.I. 2.61–3.78) and kidney disease (IRR: 2.05; 95% C.I. 1.66–2.53) increased the risk of death among patients hospitalized with HZ. In the presence of comorbidities, males had a higher risk of death than females, and age was the strongest predictor. The risk increased significantly after the age of 40, reaching an IRR of 37.16 in patients over 80 years old with neoplasms.

**Conclusions:**

The findings of this Real-World study may raise awareness of the risks associated with HZ and support vaccination efforts to prevent infection, particularly among older adults and individuals with comorbidities.

**Supplementary information:**

The online version contains supplementary material available at 10.1186/s12879-025-12319-x.

## Background

The varicella-zoster virus (VZV) is a neurotropic human herpesvirus responsible for the primary infection that causes chickenpox, one of the most contagious human diseases [1], characterized by viremia, widespread skin rash, and dissemination to multiple sensory ganglia where the virus remains latent for life. Reactivation of the latent virus, years or even decades after the primary infection, leads to the typical clinical manifestation known as herpes zoster (HZ). By the age of 15 years, over 90% of the population has been infected by varicella in European countries before the widespread implementation of varicella vaccination programs [2].

In Italy, the annual incidence of HZ is 6.3 per 1,000 person-years, with 73% of cases occurring in adults [3]. The overall HZ incidence rate per 1000 person-years (PY) was also estimated as 6.46 (95% CI: 5.99–6.95), increasing with age to 9.12/1000 PY (95% CI: 7.50–10.99) in 75–79-year-olds [4].

Numerous epidemiological studies have shown that the incidence of HZ increases significantly after the age of 50, due to the natural decline of VZV-specific cell-mediated immunity (CMI). Regardless of age, immunocompromised individuals, those with neoplasms, autoimmune diseases, diabetes, chronic kidney disease, and chronic obstructive pulmonary disease (COPD), have an increased risk of viral reactivation due to reduced immune response [5–9].

Several population-based studies have shown that individuals with chronic diseases, especially diabetes and cardiovascular disorders, have a higher risk of developing HZ compared with healthy adults, and an even higher risk of hospitalization and severe complications [8, 10, 11]. Herpes zoster (HZ) can cause a wide range of complications, including postherpetic neuralgia, ocular involvement, bacterial superinfections, neurological manifestations, and hospitalizations, particularly among the elderly and immuno-compromised patients [10, 11]. The risk of severe HZ and its related complications increase in the presence of comorbidities such as diabetes mellitus, cardiovascular diseases, chronic obstructive pulmonary disease (COPD), chronic kidney disease, and oncological conditions [6, 11]. Epidemiological studies indicate that hospitalization rates for HZ are higher in individuals with these comorbidities, and the disease represents a significant burden on the healthcare system, both in terms of direct costs and long-term management of complications [12]. These findings highlight the importance of understanding how comorbid conditions influence the clinical course and outcomes of HZ, particularly in aging populations. In Italy, as in many other countries with a rapidly aging population, the impact of HZ is expected to increase in the coming decades [10].

As shown in previous clinical trials and real-world studies, a safe and effective vaccine is available. [12, 13] Still, this has not yet resulted in adequate vaccination coverage and HZ continues to represent a significant burden of disease in the frail adult population, leading to continued high incidence, complications (such as post-herpetic neuralgia), and avoidable hospital admissions [5, 14].

The 2023–2025 National Vaccine Prevention Plan (PNPV) recommends herpes zoster vaccination for individuals aged 65 years, and from the age of 18 for those with high-risk conditions such as diabetes mellitus, cardiovascular disease (excluding isolated hypertension), chronic obstructive pulmonary disease (COPD) or bronchial asthma, congenital or acquired immunodeficiency or those undergoing immunosuppressive therapy, chronic renal failure or dialysis and those with current or particularly severe forms of herpes zoster [15–17]. Currently, only the recombinant vaccine (RZV) is available in Italy, and the coverage target for the 65-year-old cohort ( > 50%) is still far from being reached (around 9%) [15, 17]. Monitoring disease trends and burden remains essential to guide public health strategies effectively [18].

In this context, Hospital Discharge Records (HDR) represent a valuable source of real-world data, useful to evaluate the trends in hospitalizations and the clinical severity of herpes zoster infections over time [10]. Although the link between comorbidities and the increased risk of herpes zoster is well documented, little is known about the strength of the association between specific pathological conditions and the risk of in-hospital mortality [14, 19]. This aspect is particularly relevant in light of increasing life expectancy and the growing prevalence of chronic diseases [3]. Previous studies at the regional level have provided partial data, but systematic analyses at the national level covering a broad time period are still lacking [20].

The primary objective of this study is to assess the burden of hospitalizations related to herpes zoster in Italy between 2011 and 2023. The study aims to describe the epidemiological impact of HZ at the national level, with particular attention to the distribution of comorbidities and their contribution to an increased risk of mortality.

## Methods

### Study design and methods

This is a retrospective population-based study, conducted among all patients hospitalized with HZ in Italy, between January 1^st^ 2011 and December 31^st^, 2023.

The data source was the Italian Hospital Discharge Database (HDD) obtained from Ministry of Health (General Directorate for Health Planning, VI Office - Monitoring and evaluation of the Essential Levels of Care (LEA) and regional Recovery Plans within the Italian National Health Service). This database contains administrative and health data regarding hospital admissions, that all public and privately-owned hospitals in Italy are legally required to report. For each admission, a primary diagnosis (PD) is reported, i.e. the clinical condition which took up the greatest amount of resources and therefore involved the greatest cost for the hospital. Up to three additional secondary diagnosis (SD) may be listed. The clinical information is coded by the international ICD-9-CM system (International Classification of Diseases, 9th revision, clinical modification), currently used in Italy.

A retrospective analysis of HDD was performed to extract all hospitalizations carried out between 2011 and 2023 in Italy, bearing ICD9-CM codes related to HZ infection in primary or secondary diagnosis. All hospital discharge records (HDRs) containing HZ diagnostic codes, whether in the primary or secondary diagnoses, were included for each year. In cases where a patient had multiple admissions for the same condition, only the first hospitalization was counted, and any subsequent admissions for the same patient with HZ codes were excluded.

Hospitalizations with HZ were obtained by selecting any of the four fields of the HDR diagnosis codes classified as follows: HZ with meningitis, HZ with other nervous system complications, HZ with post-herpetic trigeminal neuralgia- polyneuropathy, HZ with ophthalmic complications, HZ with other specified complications, HZ with unspecified complication, HZ without complication (Table 1).

**Table 1:** ICD-9-CM codes used to identify HZ-related hospitalizations

ICD-9-CM Codes	Diagnosis	Diagnosis Group
053.0	Herpes zoster with meningitis	HZ with meningitis
053.1	Herpes zoster with other nervous system complications	HZ with other nervous system complications
053.10	Herpes zoster with unspecified nervous system complication	
053.11	Geniculate herpes zoster	
053.19	Other nervous system complications	
053.12	Post-herpetic trigeminal neuralgia	HZ with post-herpetic trigeminal neuralgia- polyneuropathy
053.13	Post-herpetic polyneuropathy	
053.2	Herpes zoster with ophthalmic complications	HZ with ophthalmic complications
053.20	Herpes zoster dermatitis of eyelid/herpes zoster ophthalmicus	
053.21	Herpes zoster keratoconjunctivitis	
053.22	Herpes zoster iridocyclitis	
053.29	Other	
053.7	Herpes zoster with other specified complications	HZ with other specified complications
053.71	Otitis externa due to herpes zoster	
053.79	Other specified complications	
053.8	Herpes zoster with unspecified complication	HZ with unspecified complication
053.9	Herpes zoster without mention of complication	HZ without complication

To identify comorbidities, patients with the presence of malignant and benign tumors (ICD-9-CM codes: 140–208.9 malignant neoplasms, 210–229.9 benign tumors, 230–234.9 carcinoma in situ, 235–238.9 neoplasms of uncertain behavior, 239–239.9 neoplasms of unspecified nature), chronic obstructive pulmonary disease – COPD (ICD-9-CM: 490–496), kidney diseases (ICD-9-CM: 580.0–589.9 nephritis, nephrotic syndrome, and nephrosis; 590–599.9 other diseases of the urinary system), diabetes mellitus (ICD-9-CM: 250.0–250.93), and autoimmune diseases (ICD-9-CM: 695.4 localized lupus erythematosus, 710.0 systemic lupus erythematosus, 710.2 Sjögren’s syndrome, 714.0–714.9 rheumatoid arthritis and other inflammatory polyarthropathies), all of which are known to induce a reduced VZV-specific cell-mediated immune response, were considered. Diseases included in Charlson Comorbidity Index and related ICD-9-CM codes were considered as shown in Table 2:

**Table 2:** Diseases included in Charlson comorbidity index and related ICD-9-CM codes

Type of comorbidity	Codes
Malignant neoplasms	140–208.9
Chronic Obstructive Pulmonary Disease - COPD	490–496
Kidney diseases and other diseases of the urinary system	580.0–589.9 nephritis, nephrotic syndrome, and nephrosis 590–599.9 other diseases of urinary system
Diabetes mellitus	250.0–250.93
Autoimmune diseases	695.4 localized lupus erythematosus 710.0 systemic lupus erythematosus 710.2 Sjogren’s syndrome 714.0–714.9 rheumatoid arthritis and other inflammatory polyarthropathies

### Statistical analysis

#### Descriptive analyses

Descriptive analyses were used to illustrate the characteristics (sex, age classes, length of stay, complications and co-morbidities) of HZ associated hospital admissions. The discrete and nominal variables (sex, age classes, complications and co-morbidities) were described through frequencies and percentages; the quantitative variables (length of stay) were expressed in terms of mean and standard deviation.

#### Herpes zoster hospitalization rates by age groups: ≤19, 20–39, 40–59, 60–79, ≥80

The annual hospital admission rates for HZ, expressed as person-year, as well as stratified by age groups, were calculated by dividing the annual number of hospital admission for HZ extracted from the hospital discharge database for the annual resident population stratified by age groups: ≤19, 20–39, 40–59, 60–79, ≥80 and sex. The resident population was obtained by the Italian Institute of Statistics (ISTAT) website (per 100,000 inhabitants) for the period 2011–2023 [21]. The average length of stay was calculated for all cases and for gender. The average annual percentage change (AAPC) was calculated to highlight any significant change in time trends for overall hospitalization rates and stratified for sex [22].

#### Analysis of risk factors associated to herpes zoster hospitalizations in the entire Italian general population

Poisson regression models, adjusted per age classes and sex, were used to identify factors associated with an increased risk of death, with associations reported as Incidence Rate Ratios (IRRs) and 95% confidence intervals (95% CIs). The number of hospitalizations with and without the comorbidity was included as an offset in the model. As per the Poisson model assumptions, the count of deaths was used as the dependent variable. For each year of the study period. Each subject was considered exposed for the entire year of the study period.

The IRRs were used to compare incidence rates (IRs) between classes of each explanatory categorical variable. The statistical package STATA used as denominator for the calculation of IRR the IR of the lowest coded class of the categorical variable of interest. A p-value of < 0.05 was the criterion for statistical significance. Data analysis was performed using STATA/BE 18.0.

#### Sensitivity analysis

To assess the robustness of our results, we conducted a sensitivity analysis including only hospitalizations with herpes zoster listed as the principal diagnosis. All primary outcomes and analyses were repeated in this restricted cohort. Comparisons between the main analysis and sensitivity analysis (with HZ in PD) were performed to evaluate potential bias due to including HZ in all diagnoses.

### Study population

To evaluate the epidemiological burden of HZ, HDRs with HZ between 2011 and 2023, with or without complications, were extracted from the national HDD. The characteristics of hospitalizations were described by age groups: ≤19, 20–39, 40–59, 60–79, ≥80.

Data provided by the Ministry of Health did not contain any patient identifiers and were therefore completely anonymous. Hence, a patient’s consent was not required. The request for evaluation of this observational study has been approved by the Internal Review Board (University of L’Aquila, protocol number 20338, dated 16 February 2022).

## Results

Between 2011 and 2023, a total of 47,933 hospitalizations with HZ were recorded, with an annual average of approximately 3,687 cases, equivalent to about 10 hospitalizations per day.

The distribution of hospitalization rates for HZ showed a significant downward trend, decreasing from 8.03 per 100,000 inhabitants in 2011 to 5.18 per 100,000 in 2023. This decline was particularly marked during the 2019–2021 period, dropping from 6.16 in 2019 to 4.32 in 2020 and 4.29 in 2021.

**Fig. 1:**
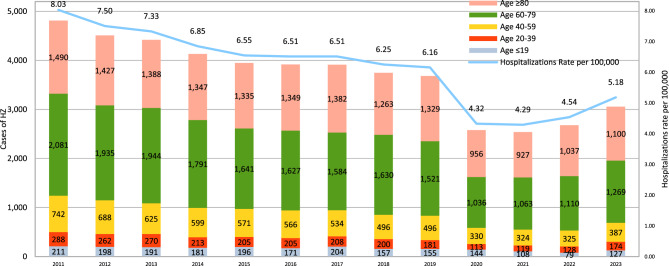
Temporal trend of hospitalizations and number of hospitalized cases per age classes and years

Regarding the age distribution, across all years analyzed, the highest number of cases occurred in the 60–79 age group, with a peak of 2,081 hospitalizations in 2011 and a minimum of 1,036 in 2020. Among individuals aged 80 and over, a progressive decline in hospitalizations was observed—from 1490 cases in 2011 to 927 in 2021—followed by a slight increase in 2022 and 2023 1,037 and 1,100 cases respectively (Fig. 1).

**Fig. 2:**
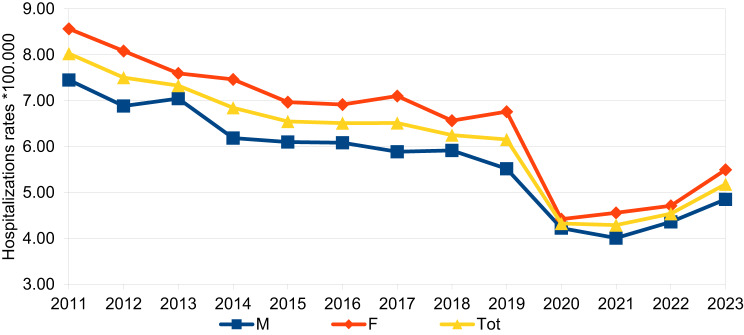
Temporal trend in hospitalization rates by gender and year

**Fig. 3:**
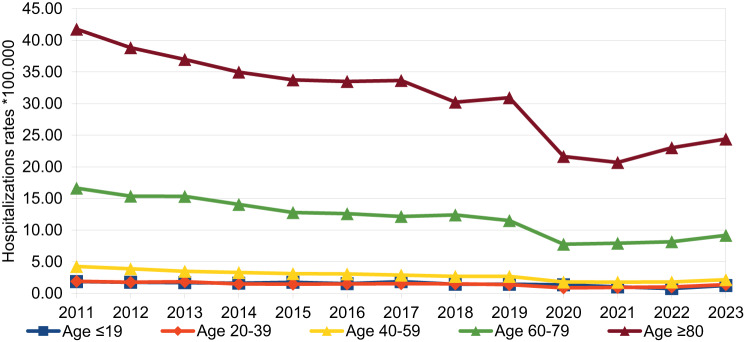
Temporal trend in hospitalization rates by age group and year

The temporal trend of hospitalization rates by age group, sex and years is presented in Figs2 and 3 and it is in line with the trend in number cases of HZ hospitalized per age classes and years. The Average Annual Percentage Change (AAPC) in overall hospitalization rates, used to highlight any significant change in time trend, stratified by age group and sex, from 2011 to 2023, is reported in Table 3. The overall AAPC was −4.73%, indicating an average annual decrease in hospitalization rates of 4.73%. When stratified by sex, a statistically significant reduction was observed. A statistically significant reduction was found for male, − 4.60% (95% CI:-5.81%; −3.37%), and female, −4.87% (95% CI:-6.39%; −3.32%). AAPC for age was calculated for the five age groups (≤19, 20–39, 40–59, 60–79, ≥80) and showed a statistically significant decrease, with values of −5.11% (95% CI: −7.37%; −2.85%), −4.86% (95% CI: −7.09%; −2.63%), −6.82% (95% CI: −8.28%; −5.35%), −6.06% (95% CI: −7.48%; −4.63%), and −5.28% (95% CI: −6.61%; −3.95%), respectively. This indicated a greater reduction in hospitalization rates in the 40–59 age group and the 60–79 age group.

**Table 3:** Average annual percentage change (AAPC) in overall hospitalization rates and stratified for age classes and sex of the hospitalized patients. From 2011 to 2023

	Overall AAPC (95% CI)
**Total**	−4.73% (−6.09%; −3.36%) *
**Sex**	
Male	−4.60% (−5.81%; −3.37%)*
Female	−4.87% (−6.39%; −3.32%)*
**Age classes**	
≤19	−5.11% (−7.37%; −2.85%)*
20–39	−4.86% (−7.09%; −2.63%)*
40–59	−6.82% (−8.28%; −5.35%)*
60–79	−6.06% (−7.48%; −4.63%)*
≥80	−5.28% (−6.61%; −3.95%)*

*Statistically significant trend

AAPC: Average annual percentage change

The average length of stay was 10.92 ± 10.75 days, increasing with age: from 7.61 days in patients aged ≤19 years to 12.17 days in those over 80 years (data not shown in the table).

The distribution of the absolute and percentage frequency of hospitalized patients by sex, age classes, and presence of comorbidities was stratified by Alive/Dead status and is shown in Table 4. The statistical significance of the comparisons was assessed using the chi-square test. Males accounted for 45.23% (21,678) of the sample, while females represented 54.77%. The most represented age group was 60–79 years, accounting for 42.21% of hospitalizations, and 76.28% of hospitalizations involved individuals aged 60 years and older. The highest frequency of deaths was observed among patients aged over 80 (66.17%), followed by the 60–79 age group (26.88%). A total of 13,280 hospitalizations (27.71%) involved patients with comorbidities. Considering that each patient may have more than one diagnosis, the number of comorbid conditions rose to 14,522. HZ was associated with malignant neoplasms, COPD, kidney diseases, diabetes mellitus, and autoimmune diseases in 32.76% (4,757), 16.07% (2,334), 21.59% (3,136), 24.96% (3,624), and 4.62% (671) of cases, respectively. The presence of comorbidities was associated with a higher frequency of death, with malignant neoplasms being the most significant (48.51%), followed by kidney diseases (34.65%), chronic obstructive pulmonary disease – COPD (8.91%), diabetes mellitus (6.60%), and autoimmune diseases (1.32%) (Table 4).

**Table 4:** Main characteristics of subjects discharged with an HZ diagnosis by Alive/Dead (Italy, 2011–2023)

	Total	Alive, n (%)	Dead, n (%)	p-value #
	47,933	47,256 (98.59)	677 (1.41)	
**Sex**, n (%)				0.219
Male	21,678 (45.23)	21,356 (45.19)	322 (47.56)	
Female	26,255 (54.77)	25,900 (54.81)	355 (52.44)	
**Age classes**, n (%)				< 0.001
≤19	2,122 (4.43)	2.120 (4.46)	2 (0.30)	< 0.001
20–39	2,566 (5.35)	2,562 (5.42)	4 (0.59)	< 0.001
40–59	6,683 (13.94)	6,642 (14.06)	41 (6.06)	< 0.001
60–79	20,232 (42.21)	20,050 (42.43)	182 (26.88)	< 0.001
≥80	16,330 (34.07)	15,882 (33.61)	448 (66.17)	< 0.001
Number of comorbidity diagnosis*, n (%)	14,522	14,219	303	
Malignant neoplasms*	4,757 (32.76)	4610 (32.42)	147 (48.51)	< 0.001
Chronic Obstructive Pulmonary Disease- COPD*	2,334 (16.07)	2,307 (16.22)	27 (8.91)	0.283
Kidney diseases*	3,136 (21.59)	3,031 (21.32)	105 (34.65)	< 0.001
Diabetes mellitus*	3,624 (24.96)	3,604 (25.35)	20 (6.60)	< 0.011
Autoimmune diseases: systemic lupus erythematosus - Rheumatoid arthritis - Sjogren’s syndrome*	671 (4.62)	667 (4.69)	4 (1.32)	0.071

*n (%) data referring to the number of diagnoses (a single hospitalized may present with more than one comorbidity)

# chi-square test

Complicated HZ was diagnosed in 49.73% of cases (23,838). The most frequent complications were neurological 46.27% (11.413), followed by ophthalmic 23.69% (5,843) and other specified complications 18.50% (4,562). The remaining 11.55% of cases (2,848) involved HZ with other unspecified complications. (data not shown in the tables).

The data obtained in this study analyzed the factors associated with an increased risk of death in the presence of comorbidities. The main comorbidities identified were reported in the Table 5, in order of frequency.

**Table 5:** Distribution of comorbidities by frequency

Malignant neoplasms*	4,757 (32.76)
Diabetes mellitus*	3,624 (24.96)
Kidney diseases*	3,136 (21.59)
Chronic Obstructive Pulmonary Disease- COPD*	2,334 (16.07)
Autoimmune diseases: systemic lupus erythematosus - Rheumatoid arthritis - Sjogren’s syndrome*	671 (4.62)

*n (%) data referring to the number of diagnoses (a single hospitalized may present with more than one comorbidity)

The multiple Poisson regression analysis highlighted that hospitalized patients with malignant neoplasms have a 3.15 times higher risk of death compared to those without malignant neoplasms (IRR 3.15, 95% CI 2.61–3.78). Among patients with neoplasms, males had a 1.24 times higher risk of death compared to females (IRR 1.24, 95% CI 1.07–1.45). In this subgroup, the risk increases significantly with age starting from 40 years: age group 40–59: IRR 7.46, 95% CI 2.66–43.15, age group 60–79: IRR 10.71, 95% CI 2.66–43.15, age group ≥80: IRR 37.16, 95% CI 9.26–149.18.

The presence of COPD was not significantly associated with an increased risk of death. However, the risk remained higher among males (IRR 1.30, 95% CI 1.12–1.52) and in individuals aged ≥40 years: age group 40–59: IRR 6.64, 95% CI 1.60–27.43, age group 60–79: IRR 9.89, 95% CI 2.45–39.82, age group ≥80: IRR 31.13, 95% CI 7.76–124.91 (Table 5).

Hospitalized patients with kidney diseases had a 2.05 times higher risk of death compared to HZ patients without kidney diseases (IRR 2.05, 95% CI 1.66–2.53). The risk of death increased in males (IRR 1.26, 95% CI 1.08–1.48) and with age: age group 40–59: IRR 6.39, 95% CI 1.55–26.44, age group 60–79: IRR 9.23, 95% CI 2.29–37.19, age group ≥80: IRR 27.93, 95% CI 6.96–112.08 (Table 5).

The presence of autoimmune diseases was not significantly associated with an increased risk of death. However, in males (IRR 1.28, 95% CI 1.11–1.50) and in individuals over 40, the risk of death was higher than expected: age group 40–59: IRR 6.60, 95% CI 1.60–27.29, age group 60–79: IRR 9.67, 95% CI 2.40–38.97, age group ≥80: IRR 30.24, 95% CI 7.54–121.32 (Table 6).

**Table 6:** Results of multiple Poisson regression models. Analysis to identify factors associated with an increased risk of death due to the presence of comorbidities

Presence of co-morbidity	n (%)	IRR	95% C.I.	p-value
**Malignant neoplasms**		3.15	2.61–3.78	** < 0.001**
**Gender**		1.24	1.07–1.45	
Male vs. Female	2,458 (51.67) vs. 2299 (48.33)			**0.005**
**Age groups (years)**				
≤19^a^	388 (8.16)	1		
20–39	179 (3.76)	2.02	0.36–11.01	**0.418**
40–59	729 (15.32)	7.46	1.80–30.86	**0.006**
60–79	2,446 (51.42)	10.71	2.66–43.15	**0.001**
≥80	1,015 (21.34)	37.16	9.26–149.18	** < 0.001**
**COPD**		0.64	0.44–1.09	**0.088**
**Gender**		1.30	1.12–1.52	
Male vs. Female	1,265 (54.20) vs. 1069 (45.80)			**0.001**
**Age groups (years)**				
≤19^a^	4 (0.17)	1		
20–39	16 (0.69)	1.66	0.30–9.07	**0.589**
40–59	124 (5.31)	6.64	1.60–27.43	0.009
60–79	1,144 (49.01)	9.89	2.45–39.82	**0.001**
≥80	1,046 (44.82)	31.13	7.76–124.91	** < 0.001**
**Kidney diseases**		2.05	1.66–2.53	** < 0.001**
**Gender**				
Male vs. Female	1,625 (51.82) vs. 1511 (48.18)	1.26	1.08–1.47	**0.003**
**Age groups (years)**				
≤19^a^	33 (1.05)	1		
20–39	73 (2.33)	1.63	0.30–8.93	**0.570**
40–59	288 (9.18)	6.39	1.55–26.44	**0.010**
60–79	1,212 (38.65)	9.23	2.29–37.19	**0.002**
≥80	1,530 (48.79)	27.93	6.96–112.08	** < 0.001**
**Diabetes mellitus**		0.34	0.22–0.53	** < 0.001**
**Gender**		1.30	1.12–1.52	
Male vs. Female	1,775 (48.98) vs. 1849 (51.02)			**0.001**
**Age groups (years)**				
≤19^a^	10 (0.28)	1		
20–39	24 (0.66)	1.66	0.30–9.09	**0.556**
40–59	365 (10.07)	6.82	1.65–28.22	**0.008**
60–79	1,948 (53.75)	10.32	2.56–41.57	**0.001**
≥80	1,277 (35.24)	31.95	7.96–128.20	** < 0.001**
**Autoimmune diseases**		0.59	0.22–1.57	**0.290**
**Gender**		1.28	1.11–1.50	
Male vs. Female	132 (19.67) vs. 539 (80.33)			**0.001**
**Age groups (years)**				
≤19^a^	38 (5.66)	1		
20–39	61 (9.09)	1.66	0.30–9.08	**0.557**
40–59	145 (21.61)	6.60	1.60–27.29	**0.009**
60–79	301 (44.86)	9.67	2.40–38.97	**0.001**
≥80	126 (18.78)	30.24	7.54–121.32	** < 0.001**

IRR: incident rate ratios adjusted for age and gender

^a^: reference category

### Sensitivity analysis

When restricting the analysis to cases with HZ as the principal diagnosis (*n* = 21,919) the overall trends and associations remained consistent with the main analysis. The Poisson regression model confirmed that comorbidities contributing to an increased risk of mortality were malignant neoplasms (IRR:2.55; 95% C.I. 1.49–4.35) and kidney desease (IRR:3.01; 95% C.I. 1.67–5.39). These results are in line with the main analysis, confirming the robustness of the main findings. In this analysis, the comorbidity “Autoimmune disease” was not included because there were no deaths among patients with PD who had herpes zoster and this comorbidity, an association that was also non-significant in the main analysis (see the Supplementary Materials).

## Discussion

The first objective of the study was to assess this burden in Italy in the period between 2011 and 2023, thus expanding the time interval previously examined at a national level [18, 20] and extending this assessment to the more recent pandemic period, through a systematic analysis of hospitalizations with HZ using Hospital Discharge Records (HDRs), which are a robust and standardized source of real-world data.

The results showed that HZ remains a significant condition during the hospitalization of patients over 60 years old, especially those with major comorbidities. On average, 3687 patients were hospitalized annually with a diagnosis of HZ, and the annual hospitalization rate per 100,000 people ranged from a high of 8.03 in 2011 to a low of 4.29 in 2021, in line with rates in other countries [14, 23, 24].

The time trend analysis showed a statistically significant average annual decrease in hospitalisation rates of 4.73%, in line with that reported by Amodio et al. [20]. The analysis confirmed the decrease in hospital admissions, especially during the pandemic years. This trend was consistent with the data reported in the literature (typically between 4 and 5 per 100,000) and may be partly attributed to the impact of the Covid-19 pandemic [20]. However, it showed signs of gradual growth from 2022, although rates remained lower than in the pre-pandemic period. It is not easy to identify the main reasons behind this trend. However, some considerations may help to explain the possible scenarios. As with other diseases, it seems plausible that this trend can be attributed to the reduced availability of beds since 2020 rather than to an actual reduction in the burden of disease, as demonstrated by the collapse in the first year of the pandemic and the subsequent increase mentioned above [20, 25, 26]. In fact, from 2011 to 2023 in Italy, hospital admissions for all causes decreased from 14.9 to 11.8 million per year (an overall reduction of 21.09%) [27]. However, in our study, the relative reduction observed for hospitalisations due to HZ (−36.47% overall) was notably higher than that observed for total hospitalisations.

As shown in previous studies, important differences in hospitalization exist when stratifying by sex and age [1, 18, 20]. The higher prevalence of HZ found in the hospitalized female population (54.77% vs. 45.23%) could reflect different hospitalization rates between the sexes. Some authors attribute this difference to women’s greater propensity to access healthcare services and different susceptibility to varicella-zoster virus (VZV) reactivation [28]. However, it could also be due to the female sex’s greater longevity [20]. Interestingly, this difference between the sexes tends to be attenuated when considering only cases of hospitalization resulting in death. Our findings showed that males generally had a higher risk of death than females (Table 5). According to the “exogenous boosting” hypothesis, frequent exposure to the virus through contact with children with chickenpox could provide women with protective immune reinforcement, even though a recent study reported a stronger boosting effect among men than women [29–31].

Our analysis showed that the prevalence of HZ increased with age, especially after age 60 (76.28% of hospitalizations), as well as the mortality rate (93.05% of deaths occurred in people over the age of 60), even when the data were adjusted for sex and comorbidities, in line with the decline in VZV-specific cell-mediated immunity [32]. This evidence is not new [18, 22] and supports the vaccination policies in place in our country. It provides useful information for evaluating the expansion of vaccine-eligible population groups, considering that the current target is limited to the 65-year-old cohort. In fact, the progressive aging of the Italian population and the increase in chronic diseases require strengthening prevention strategies for this population group.

The second major objective of the study was to evaluate the strength of the association between groups of pathological conditions and the risk of mortality during the hospitalization of patients with HZ.

We found that malignant neoplasms and renal diseases, the two disease groups most prevalent in the group of hospitalized patients with HZ who died (48.51% and 34.65%, respectively), also proved to be the comorbidity groups with a stronger association with the risk of death in these patients, respectively with 3.15 and 2.05 times higher than in the hospitalized population with HZ without these comorbidities, even adjusting for age and sex (*p* < 0.001). This finding is consistent with the PNPV indications and is due to the substantial immunodeficiency experienced by these patients. Moreover, the manifestation of HZ in these patients could even represent an early marker of malignancy [8, 11, 33].

It is more difficult to explain why diabetes mellitus was statistically significantly associated with a 66% reduction in mortality (Table 5). The data available in the literature do not show a consistent increase in the risk of mortality among patients hospitalized for herpes zoster with diabetes. Most studies have focused on the increased incidence of HZ in diabetic individuals rather than on mortality outcomes once the infection has occurred [1, 34, 35]. This is consistent with our data, which show that diabetes was the second most frequent diagnosis of comorbidity (*n* = 3,624, 24,96%). Studies investigating the main causes of death among individuals with diabetes have identified other pathological conditions such as cardiovascular diseases (CVD) [36, 37]. It is therefore plausible that the inverse association observed in our analysis does not reflect a protective effect, but rather differences in hospitalization patterns or baseline clinical characteristics. In our study, the inverse association between diabetes and mortality remained consistent in the sensitivity analyses (restricted to cases with a primary diagnosis), suggesting that the result was not due to residual confounding. However, this finding should be interpreted with caution, as unmeasured variables and the aggregated nature of the data may have influenced the observed effect.

Additionally, the absence of a positive association with mortality should not lead one to underestimate the impact of diabetes on hospitalizations, including readmissions and complications. In general, diabetes contributes to the severity and complexity of the clinical course [6].

Further studies based on individual-level data and more complex multivariate models will be necessary to clarify the relationship between diabetes and mortality in patients hospitalized for HZ.

Our findings support the need to increase vaccine coverage among the Italian target population (which has not been evaluated here), particularly among older age groups and individuals with comorbidities resulting in immunodeficiency, to prevent HZ and its complications. The availability of anti-zoster vaccines is an important tool for protecting citizens’ health and ensuring the financial sustainability of the Italian National Health Service thanks to their favourable cost-benefit profile [3, 12, 13, 22, 35].

Despite this, vaccination coverage in Italy remains extremely low, according to a survey of 10,000 people [17]. Herpes zoster continues to have a significant clinical, social, and economic impact, suggesting the urgent need for health strategies that promote vaccination and eliminate organizational, informational, and economic barriers to access [38].

## Limitations of the study

Despite the obvious merits of a study based on real-world data, it is important to note that it is not without its limitations. Some methodological limitations related to the use of HDRs as the primary source of data must be considered. HDRs are administrative tools designed primarily for management and reimbursement purposes rather than for systematically collecting in-depth clinical data such as disease severity, vaccination status, symptoms duration, treatments received and patients’ immune profiles [39].

Nevertheless, the Italian HDD had an average completeness level of 98.3% between 2011 and 2023 [40]. Therefore, the quality of the database is extremely high, as previous studies have highlighted [38].

To reduce the risk of duplicate counting, hospitalizations in which the same patient had already been hospitalized for HZ were excluded. The analysis is based only on cases that required hospitalization, and it completely excludes mild forms or those managed in outpatient settings. This results in a partial view of the real impact of HZ on the general population. Additionally, the absence of some potentially confounding variables, such as socioeconomic status, level of territorial care, and number of healthcare accesses for diabetes-related complications (e.g., cardiovascular) [6] may have influenced the observed associations between clinical conditions and outcomes by introducing uncontrollable confounding elements. In Italy, HDRs are still coded using ICD-9-CM; however, potential variability in coding accuracy and hospital admission criteria across time and institutions may have influenced the observed trends.

Another limitation of the study is the absence of a comparison between hospitalized patients with and without HZ. Such a comparison would provide insight into the true extent of the association between comorbidity groups, HZ, and mortality. Future studies should verify the hypothesis that the association between mortality and malignancies or renal disease is stronger in the HZ population than in the non-HZ population.

As we did not evaluate the effectiveness of the HZ vaccine or the Italian vaccination coverage, future studies should also determine its impact on hospitalization trends, particularly since the introduction of the vaccine in the active supply of the Italian vaccine calendar in 2017. However, this will be challenging since Italy does not currently have a national registry of vaccine coverage for HZ vaccine.

Finally, it will be necessary to extend the monitoring of hospitalization trends for HZ to verify whether they will continue to decline or if the increase in cases recorded in 2023 will rise beyond pre-pandemic levels.

Despite these limitations, our work significantly contributes to understanding the impact of herpes zoster in Italy by offering an estimate of the hospitalization burden from 2011 to 2023. By analyzing temporal trends and identifying some of the main factors associated with an increased risk of mortality, including the most relevant comorbidity groups, the study provides valuable insights to inform health planning and preventive strategies, particularly for vulnerable populations.

## Conclusions

The results of this Real-World Medicine study, which aimed to evaluate the association between comorbidities and HZ infection, show an overall decrease in the hospital burden during the study period, with a particularly marked decline between 2019 and 2021, attributable to the Covid-19 pandemic, followed by an increase in more recent years. The reduction in hospitalizations was more pronounced among women than men.

In patients hospitalized for HZ, the presence of comorbidities, such as malignant neoplasms and kidney diseases, represents a factor associated with a higher risk of adverse health outcomes compared to patients without such conditions. These findings highlight the need for particular attention in managing the most fragile patients and for strengthening preventive strategies in their favor. Since HZ is a vaccine-preventable infection, it is essential to reinforce public health actions aimed at preventing infection and reducing the clinical burden in patients with comorbidities [41–47].

In this context, the involvement of treating specialists is crucial to systematically integrate vaccination schedules into patients’ diagnostic and therapeutic care pathways. This approach ensures targeted and personalized interventions, consistent with a patient-centered care model, as emphasized in the PNPV [16].

## Electronic supplementary material

Below is the link to the electronic supplementary material.

Supplementary Material 1

## Data Availability

The data that support the findings of this study are available from Italian Ministry of Health, but restrictions apply to the availability of these data, which were used under license for the current study, and so are not publicly available.

## Abbreviations

VZV: Varicella-zoster virus
PD: Primary diagnosis
SD: Secondary diagnosis
HZ: Herpes Zoster
HDRs: Hospital Discharge Records
HDD: Hospital Discharge Database
ICD-9-CM: International Classification of Disease, 9^th^ Revision, Clinical Modification
PNPV: National Vaccination Prevention Plan
RZV: Recombinant adjuvanted vaccine
ZVL: Live attenuated vaccine
