# Supplementary material for: Comorbidities in hospitalized patients with herpes zoster: an Italian retrospective observational study in the years 2011–2023
Source: BMC Infect Dis. 2025 Dec 25;26:185. doi: 10.1186/s12879-025-12319-x (PMC12849282; doi:10.1186/s12879-025-12319-x)
Supplement: Supplementary file 1 — Supplementary Material 1 [file 12879_2025_12319_MOESM1_ESM.docx]

**Supplementary Materials**

**Figure 4.** Temporal trend hospedalisations and number cases hospitalized in primary diagnosis for HZ per age classes and years.

**Figure 5.** Temporal trend in hospitalisation, for primary diagnosis for HZ, rates by gender and year


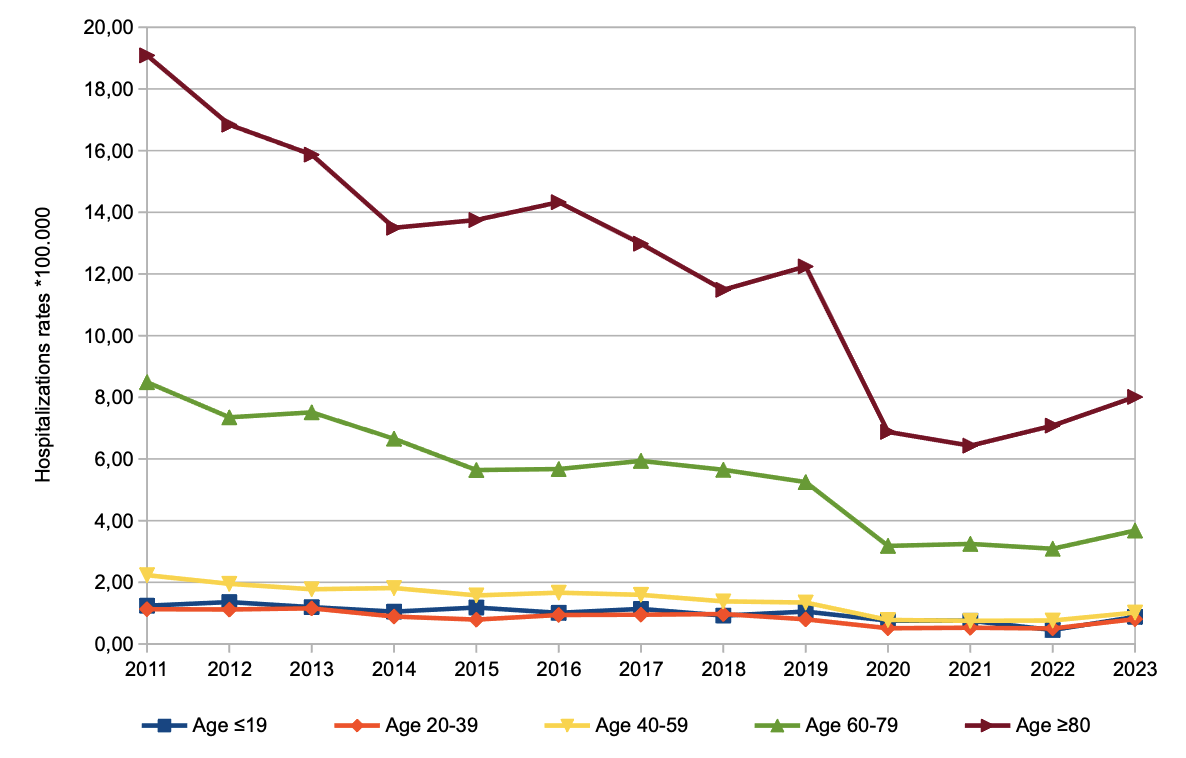


**Figure 6.** Temporal trend in Hospitalisation rates for primary diagnosis by age group and year.

| **Table 7.** Main characteristics of subjects discharged with a primary diagnosis of Herpes Zoster, by Alive/Dead (Italy 2011–2023) | | | | |
| --- | --- | --- | --- | --- |
|  | **Total** | **Alive, n (%)** | **Dead, n (%)** | **p-value** |
|  | **21,919** | **21,808 (99.49)** | **111 (0.51)** |  |
| **Sex**, n (%) |  |  |  | 0.694 |
| Male | 10,281 (46.9) | 10,231 (46.9) | 50 (45.0) |  |
| Female | 11,638 (53.1) | 11,577 (53.1) | 61 (55.0) |  |
| **Age classes**, n (%) |  |  |  | **<0.001** |
| ≤19 | 1,426 (6.51) | 1,426 (6.54) | 0 (0.00) |  |
| 20-39 | 1,532 (6.99) | 1,530 (7.02) | 2 (2.70) |  |
| 40-59 | 3,370 (15.38) | 3,367 (15.44) | 3 (2.70) |  |
| 60-79 | 9,296 (42.42) | 9,267 (42.27) | 25 (22.52) |  |
| ≥80 | 6,348 (28.97) | 6,267 (28.74) | 81 (72.97) |  |
| **Number of comorbidity diagnosis*****,** n (%) |  |  |  |  |
| Malignant neoplasms* | 1,359 (6.20) | 1,343 (6.16) | 16 (14.41) | **<0.001** |
| Chronic Obstructive Pulmonary Disease- COPD* | 711 (3.24) | 707 (3.24) | 4 (3.60) | 0.830 |
| Kidney diseases* | 991 (4.52) | 978 (4.48) | 13 (11.71) | **<0.001** |
| Diabetes mellitus* | 1,854 (8.46) | 1,851 (8.49) | 3 (2.70) | **0.029** |
| Autoimmune diseases: systemic lupus erythematosus - Rheumatoid arthritis - Sjogren’s syndrome* | 334 (1.52) | 334 (1.53) | 0 (0.00) | 0.181 |
| *n (%) data referring to the number of diagnoses (a single hospitalized may present with more than one comorbidity) | | | | |

| **Table 8.** Results of multiple Poisson regression models among patients hospitalized with a primary diagnosis of Herpes Zoster. Analysis to identify factors associated with an increased risk of death due to the presence of comorbidities. | | | |
| --- | --- | --- | --- |
| **Presence of co-morbidity** | **IRR** | **95% C.I.** | **p-value** |
| **Malignant neoplasms** | 2.55 | 1.49-4.35 | **<0.001** |
| **COPD** | 1.12 | 0.41-3.055 | 0.823 |
| **Kidney diseases** | 3.01 | 1.67-5.39 | **<0.001** |
| **Diabetes mellitus** | 0.20 | 0.09-0.93 | **0.0037** |
| IRR: incident rate ratios adjusted for age and gender | | | |
